# 2,3-Bisphosphoglycerate Mutase (BPGM), a Metabolic Player Shaping Stress-Adaptive Transcriptional States in Clear Cell Renal Cell Carcinoma

**DOI:** 10.3390/cells15070633

**Published:** 2026-03-31

**Authors:** Philipp N. Becker, Vera A. Kulow, Claudia S. Czopek, Kameliya Roegner, Gohar Ter-Avetisyan, Anica Loth, Bianca Nitzsche, Cem Erdogan, Adrian Schreiber, Michael Höpfner, Michael Fähling, Robert Labes

**Affiliations:** 1 Institut für Translationale Physiologie (CCM), Charité—Universitätsmedizin Berlin, Corporate Member of Freie Universität Berlin and Humboldt-Universität zu Berlin, Charitéplatz 1, D-10117 Berlin, Germany; philipp-nils.becker@charite.de (P.N.B.); vera.kulow@charite.de (V.A.K.); claudia.s.czopek@gmail.com (C.S.C.); kameliya.roegner@charite.de (K.R.); gohar.teravetisyan@charite.de (G.T.-A.); anica.loth@vivantes.de (A.L.); cem.erdogan@charite.de (C.E.); robert.labes@charite.de (R.L.); 2 Institut für Physiologie (CCM), Charité—Universitätsmedizin Berlin, Corporate Member of Freie Universität Berlin and Humboldt-Universität zu Berlin, Charitéplatz 1, D-10117 Berlin, Germany; bianca.nitzsche@charite.de (B.N.); michael.hoepfner@charite.de (M.H.); 3 Medizinische Klinik mit Schwerpunkt Nephrologie und Internistische Intensivmedizin, Charité—Universitätsmedizin Berlin, Corporate Member of Freie Universität Berlin and Humboldt-Universität zu Berlin, Charitéplatz 1, D-10117 Berlin, Germany; adrian.schreiber@charite.de

**Keywords:** clear cell renal cell carcinoma (ccRCC), BPGM, glycolytic flux, cancer metabolism, redox homeostasis, ferroptosis, stress tolerance, epigenetic perturbation

## Abstract

**Highlights:**

**What are the main findings?**

- BPGM is consistently upregulated in human clear cell renal cell carcinoma and contributes to cellular stress tolerance.
- Perturbation of BPGM-associated metabolic regulation induces a focused stress-response program distinct from epigenetic reprogramming by HDAC inhibition.

**What are the implications of the main findings?**

- These findings suggest an intrinsic metabolic axis linking BPGM to stress-adaptive transcriptional states in ccRCC.
- Metabolic regulators such as BPGM represent potential determinants of treatment insensitivity in metabolically resilient tumors.

**Abstract:**

Clear cell renal cell carcinoma (ccRCC) is characterized by profound metabolic reprogramming and limited responsiveness to therapeutic stressors, including epigenetic modulation. How glycolytic enzymes contribute to metabolic stress tolerance in ccRCC remains incompletely understood. We investigated the role of the glycolytic enzyme 2,3-bisphosphoglycerate mutase (BPGM) using human tumor specimens, siRNA-mediated gene silencing, functional cell-based assays, and transcriptomic profiling. Epigenetic stress was induced using Vorinostat as a pan-histone deacetylase inhibitor. BPGM expression was consistently elevated in human ccRCC compared with adjacent normal kidney tissue. A498 cells exhibited high basal BPGM levels and limited sensitivity to Vorinostat, whereas BPGM depletion increased cellular stress responses and reduced proliferative capacity. Despite similar phenotypic outcomes, *BPGM* silencing and Vorinostat treatment triggered distinct transcriptional programs. While HDAC inhibition induced widespread transcriptional changes, BPGM loss elicited a focused stress-associated response, consistent with activation of the unfolded protein response, increased lipid peroxidation, and induction of ER stress-associated genes. Our data identify BPGM as a metabolic player contributing to stress-adaptive transcriptional states in ccRCC and suggest that targeting metabolic stress adaptation may complement epigenetic strategies in renal cancer.

## 1. Introduction

Clear cell renal cell carcinoma (ccRCC) is characterized by extensive reprogramming of cellular energy metabolism and redox regulation. The kidney exhibits pronounced metabolic compartmentalization that closely follows regional oxygen availability and perfusion, with oxidative metabolism prevailing in well-perfused regions and increasing reliance on glycolytic pathways in areas exposed to physiologically low oxygen tension. Perturbations of these finely tuned metabolic programs contribute to renal pathologies, including cancer, where metabolic plasticity is a defining hallmark [1,2,3].

In ccRCC, metabolic reprogramming enables tumor cells to adapt to fluctuating nutrient availability, oxidative stress, and microenvironmental constraints [4]. This adaptation is frequently driven by oncogenic signaling and loss of tumor suppressors, most notably the inactivation of the von Hippel–Lindau (VHL) gene, resulting in stabilization of hypoxia-inducible factor-1α (HIF-1α) and enhanced expression of glycolytic enzymes [5]. Consequently, ccRCC is often described as a metabolically driven malignancy rather than being defined solely by genetic alterations [6].

A central feature of this metabolic phenotype is an increased reliance on glycolysis, even under normoxic conditions, consistent with the Warburg effect [7,8]. Beyond ATP generation, glycolysis fulfils multiple essential functions in tumor cells, including the provision of biosynthetic precursors and the maintenance of redox homeostasis. In ccRCC, glycolytic reprogramming is closely linked to antioxidant defense mechanisms, including glutathione metabolism and NADPH regeneration, enabling tumor cells to effectively buffer elevated reactive oxygen species (ROS) levels and maintain redox homeostasis [9,10]. This redox-adapted metabolic state contributes to resistance against cell death and limits the efficacy of therapeutic stressors [11].

Importantly, glycolysis is not a binary process but a dynamically regulated metabolic flux. The glycolytic rate is controlled by glucose availability, oxygen tension, allosteric regulation of key enzymes, and the expression of specific metabolic modulators that fine-tune the distribution of glycolytic intermediates. Alterations in glycolytic flux therefore have direct consequences for energy balance, redox control, and downstream metabolic pathways, making flux-modulating enzymes particularly relevant for tumor cell survival [12,13,14].

One such factor is 2,3-bisphosphoglycerate mutase (BPGM), a glycolysis-modulating enzyme that enables the diversion of glycolytic intermediates through the 2,3-bisphosphoglycerate shunt. By catalyzing the formation of 2,3-bisphosphoglycerate from 1,3-bisphosphoglycerate and its subsequent conversion to 3-phosphoglycerate, BPGM establishes the Rapoport–Luebering branch of glycolysis, which is operative only in cells expressing this enzyme [15,16,17]. As a consequence, BPGM has the capacity to fine-tune glycolytic flux, thereby influencing energy balance, redox homeostasis, and the availability of downstream metabolic intermediates. While BPGM has been extensively studied in erythrocytes, where the 2,3-bisphosphoglycerate shunt plays a central role in regulating hemoglobin oxygen affinity, its expression and functional relevance in non-erythroid tissues have long been considered negligible and therefore largely unexplored. Recently, we uncovered an unexpected role of BPGM in renal tubular cells, where it contributes to protection against oxidative stress and apoptosis [18]. These findings suggest that BPGM may act as a metabolic regulator of stress resilience in renal cells beyond its classical role in erythrocytes.

Consistent with this notion, public transcriptomic and proteomic datasets indicate elevated BPGM expression in renal cancer and suggest an association with tumor aggressiveness [19,20]. Together, these observations raise the possibility that intrinsic metabolic properties of renal cancer cells, which could be shaped in part by BPGM expression, may buffer cellular stress responses.

A key interface linking metabolic state to gene expression is epigenetic regulation. Central metabolites such as acetyl-CoA, S-adenosylmethionine, α-ketoglutarate, succinate, fumarate, NAD^+^, and 2-hydroxyglutarate serve as essential cofactors or inhibitors of histone- and DNA-modifying enzymes, including histone acetyltransferases, histone deacetylases, methyltransferases, and TET dioxygenases [21,22,23]. Consequently, changes in metabolic flux directly influence the nature and extent of epigenetic modifications and transcriptional responses [24].

In line with this concept, accumulating evidence indicates that the cellular response to epigenetic perturbation critically depends on the underlying metabolic state. In ccRCC, glycolytic adaptation and robust redox control have been implicated as key determinants of limited sensitivity to histone deacetylase (HDAC) inhibition [25,26]. In this context, epigenetic modulation has emerged as a potential strategy to sensitize tumor cells to stress. Histone deacetylase inhibitors (HDACi), such as Vorinostat, have been shown to induce broad transcriptional reprogramming and restore silenced tumor suppressor pathways in multiple cancer types [27]. More generally, metabolic regulation of epigenetic responsiveness has emerged as a fundamental principle in cancer biology [24,28,29]. However, in ccRCC, clinical and preclinical responses to HDAC inhibition have been modest and highly variable [26,30], suggesting that the intrinsic metabolic properties of renal cancer cells may buffer epigenetic stress. Nevertheless, the specific metabolic regulators that confer this form of stress resilience remain poorly defined.

Based on the distinct metabolic phenotype of ccRCC, we hypothesized that perturbation of glycolytic flux influences transcriptional programs that support tumor growth, survival, and stress tolerance. Intrinsic metabolic properties of renal cancer cells may thus shape how these cells respond to cellular stress, including epigenetic perturbation. To address this concept, we compared functional and transcriptional responses induced by loss of the glycolytic flux regulator BPGM with those elicited by histone deacetylase inhibition using Vorinostat as a model epigenetic stressor. By integrating analyses of human ccRCC specimens, targeted BPGM perturbation and transcriptomic profiling in renal cancer cells, this study aimed to determine whether BPGM-associated metabolic regulation is associated with distinct stress-adaptive gene expression states in ccRCC.

## 2. Materials and Methods

### 2.1. Human Renal Tissue

Fresh-frozen clear cell renal cell carcinoma (ccRCC) tissue and adjacent normal kidney tissue were obtained from 61 patients who underwent radical nephrectomy at Charité—Universitätsmedizin Berlin, Germany, and the University of Rostock, Germany, between 2009 and 2016, as previously described in detail by Högner et al. [31]. The use of human renal normal and tumor tissue was approved by the Ethics Committee of Charité—Universitätsmedizin Berlin (application number: EA1/181/15). Protein extracts from these samples were shared and used in the present study to detect BPGM protein expression by Western blot analysis.

### 2.2. Cell Culture and Treatments

The human ccRCC cell line A498 (ATCC HTB-44; RRID:CVCL_1056) was used for all mechanistic in vitro experiments. Cells were cultured at 37 °C in a humidified atmosphere containing 5% CO_2_ and 21% O_2_ in RPMI-1640 medium supplemented with 10% fetal calf serum, 1% penicillin–streptomycin, and 1% L-glutamine. Cells were passaged at approximately 80% confluence using trypsin–EDTA and used between passages 3 and 20.

Human 2102EP testicular germ cell cancer cells (nonseminoma, teratocarcinoma, and yolk sac tumor; RRID: CVCL_C522) were kindly provided by F. Honecker (St. Gallen, Switzerland). The 2102EP cell line was used as an epigenetically sensitive reference model and cultured under identical conditions as A498 cells.

Vorinostat (suberoylanilide hydroxamic acid, SAHA; catalog #12520, Cell Signaling Technologies, Danvers, MA, USA) was dissolved in DMSO and added directly to the culture medium. For dose–response experiments, cells were treated with 2 up to 8 µM Vorinostat for 24 h. Based on these analyses, a concentration of 4 µM Vorinostat for 48 h was used for subsequent mechanistic and transcriptomic experiments. DMSO served as the vehicle control in all experiments.

### 2.3. Glucose & Lactate Measurements

Cell culture supernatants were collected and centrifuged at 1000 rpm for 1 min at room temperature to remove cellular debris. Glucose and lactate concentrations were determined using an ABL800 FLEX PLUS analyzer (Radiometer GmbH, Krefeld, Germany) according to the manufacturer’s instructions. Measured values were normalized to cell number as indicated.

### 2.4. siRNA-Mediated BPGM Knockdown

Knockdown of *BPGM* was achieved using gene-specific small interfering RNAs (siRNAs). ON-TARGETplus SMARTpool siRNAs consisting of four different siRNAs targeting human *BPGM* (catalog #L-008917-00-0005, Horizon Discovery plc, Cambridge, UK) were used at a final concentration of 25 nM. A non-targeting siRNA pool served as the control (catalog #D-001810-10-20, Horizon Discovery plc, Cambridge, UK). Transfections were performed using DharmaFECT 1 reagent (Horizon Discovery plc, Cambridge, UK) according to the manufacturer’s instructions. Briefly, siRNAs and transfection reagent were diluted separately in serum-free, antibiotic-free medium, incubated for 5 min at room temperature, combined, and incubated for an additional 20 min before addition to cells. Knockdown efficiency was verified at the mRNA level by quantitative PCR.

For experiments directly comparing metabolic and epigenetic perturbations, siRNA transfection was performed first to allow for efficient depletion of BPGM. Twenty-four hours after transfection, cells received either DMSO or Vorinostat and were cultured for an additional 48 h prior to analysis. Three experimental groups were analyzed in parallel: (i) control cells transfected with non-targeting siRNA and treated with DMSO (si-mock), (ii) cells transfected with *BPGM*-specific siRNA and treated with DMSO (si-*BPGM*), and (iii) cells transfected with non-targeting siRNA and treated with Vorinostat (VS). This experimental design ensured identical transfection procedures, vehicle exposure, and treatment windows across all analyzed groups.

### 2.5. Quantitative PCR

Total RNA was isolated and quantitative PCR was performed as previously described [32], using a CFX Connect real-time PCR detection system (Bio-Rad Laboratories, Inc., Hercules, CA, USA) and SYBR Green Master Mix (catalog #4367659, Thermo Fisher Scientific Inc., Waltham, MA, USA). Relative gene expression was calculated using the ΔΔCt method with β-actin (*ACTB*) as the reference gene. Primer sequences are provided in Supplementary Table S1.

### 2.6. Western Blotting

Western blot analysis was performed using standard procedures. Briefly, for protein extraction, kidney tissue samples were pulverized in liquid nitrogen, and cultured cells were washed with ice-cold PBS, scraped, and pelleted at 300× *g* for 5 min at 4 °C. Proteins were lysed in buffer containing 50 mM Tris (pH 6.8), 4 M urea, 1% SDS, and 12.5 mM dithiothreitol (DTT). Protein concentrations were determined by absorbance at 280 nm using a NanoDrop 2000 spectrophotometer (Thermo Fisher Scientific Inc., Waltham, MA, USA). Equal amounts of protein were separated by SDS–PAGE and transferred to Hybond-P membranes. BPGM protein was detected using a polyclonal rabbit anti-BPGM antibody (catalog #NBP1-86064, Novus Biologicals, Centennial, CO, USA). After stripping with 0.2 mol/L NaOH, membranes were reprobed with a polyclonal rabbit anti-TUBB2B antibody (catalog #TA337744, OriGene, Rockville, MD, USA) to detect tubulin as a loading control. Chemiluminescent signals were captured using a Chemostar Imager (Intas Science Imaging Instruments GmbH, Göttingen, Germany), and band intensities were quantified with Image Studio Lite software (version 5.2, LI-COR Biosciences, Bad Homburg vor der Höhe, Germany). For analysis of human tissue samples, tumor and corresponding adjacent normal samples were processed and analyzed in a strictly paired manner on the same gel. Protein signals were normalized to tubulin to control for loading variability. To ensure comparability across different gels, a pooled reference sample was included as an external standard and used for inter-gel normalization.

### 2.7. Crystal Violet Assay

Cell numbers were determined using crystal violet staining. Cells were washed with PBS and fixed with 1% glutaraldehyde, followed by incubation with 0.1% crystal violet solution for 30 min at room temperature in the dark. Excess dye was removed by repeated washing with PBS. Bound dye was solubilized with 0.2% Triton X-100, and absorbance was measured at 570 nm using a Tecan SPARK microplate reader (Tecan Group AG, Männedorf, Switzerland).

### 2.8. CellRox Staining

To assess intracellular reactive oxygen species (ROS) levels, A498 cells seeded on glass coverslips were challenged with 0.03% H_2_O_2_ for 30 min at 37 °C, washed with PBS, and incubated with 5 nM CellROX Green reagent (catalog #C10444, Thermo Fisher Scientific Inc., Waltham, MA, USA) for 30 min at 37 °C. Cells were then fixed with 4% paraformaldehyde for 10 min at 4 °C, washed with PBS, and counterstained with DAPI. Images were acquired using an Eclipse Ti2-A microscope equipped with a DS-Qi2 camera (Nikon Instruments, Tokyo, Japan). Mean fluorescence intensity per cell was quantified using Fiji software (https://fiji.sc/; accessed date: 11 September 2025).

### 2.9. Lipid Peroxidation Assay (BODIPY-C11 Staining)

Lipid peroxidation was quantified using the fluorescent probe BODIPY™ 581/591 C11 (catalog #D3861, Thermo Fisher Scientific Inc., Waltham, MA, USA) according to the manufacturer’s instructions with minor modifications. Following the indicated treatments, cells were incubated with 10 µM BODIPY-C11 in serum-free medium for 30 min at 37 °C and protected from light. After washing with PBS, fluorescence signals were measured using a Tecan Spark microplate reader (Tecan Group AG, Männedorf, Switzerland). BODIPY-C11 incorporates into cellular membranes and exhibits a shift from red to green fluorescence upon the oxidation of lipid moieties. Oxidized and reduced signals were recorded using appropriate excitation/emission settings, and lipid peroxidation was expressed as the ratio of oxidized (green) to reduced (red) fluorescence. To account for differences in cell number, fluorescence ratios were normalized to DAPI signal intensity.

### 2.10. Next Generation Sequencing (NGS)

Total RNA from *n* = 6 independent biological replicates per condition was isolated and subjected to bulk RNA sequencing by QIAGEN Genomic Services (Hilden, Germany). Libraries were prepared using the QIASeq UPX 3′ Transcriptome Kit incorporating unique molecular identifiers (UMIs). Sequencing was performed on an Illumina NextSeq 500 platform (Illumina, Inc., San Diego, CA, USA). Reads were processed using CLC Genomics Workbench (v21.0.4), including UMI deduplication and adapter trimming, and mapped to the human reference genome (GRCh38) with ENSEMBL annotation. Differential gene expression analysis was performed using DESeq2 (version 1.48.1) [33], excluding genes with fewer than five total counts across all samples. The RNA sequencing data have been deposited in the Gene Expression Omnibus (GEO) under accession number GSE319257.

### 2.11. Functional Enrichment Analysis

Pathway enrichment analyses were performed using MSigDB Hallmark and WikiPathways gene sets. In addition, gene sets derived from epigenetic modifications annotated in the EpiFactors database [34,35] were used for targeted analysis. Gene set variance analysis (GSVA) and gene set enrichment analysis (GSEA) were conducted using the GSVA and clusterProfiler R packages (version 4.16.0) [36]. Detailed parameters are provided in Supplementary Table S2.

### 2.12. Statistical Analysis

Statistical analyses were performed using GraphPad Prism (version 9, GraphPad Software, Boston, MA, USA). Outliers were identified using the ROUT method with a Q value of 5% [37]. Data distribution was assessed using the Kolmogorov–Smirnov test. Statistical tests were selected individually for each dataset based on data distribution and variance characteristics. For comparisons between two groups, Student’s *t*-test (standard deviation difference < two-fold) or Welch’s *t*-test (standard deviation difference > two-fold) was applied for normally distributed data, whereas the Mann–Whitney U test was used for non-normally distributed data. For datasets comprising more than two groups, one-way ANOVA followed by Tukey’s post hoc test was used when data were normally distributed and standard deviations differed by less than twofold. If variances were unequal (standard deviation difference > twofold), Brown–Forsythe ANOVA with Dunnett’s T3 post hoc test was applied. For non-normally distributed datasets with more than two groups, the Kruskal–Wallis test followed by Dunn’s post hoc test was used. All statistical tests were two-sided. A *p*-value < 0.05 was considered statistically significant.

## 3. Results

### 3.1. BPGM Is Consistently Upregulated in Human Clear Cell Renal Cell Carcinoma

**Figure 1:**
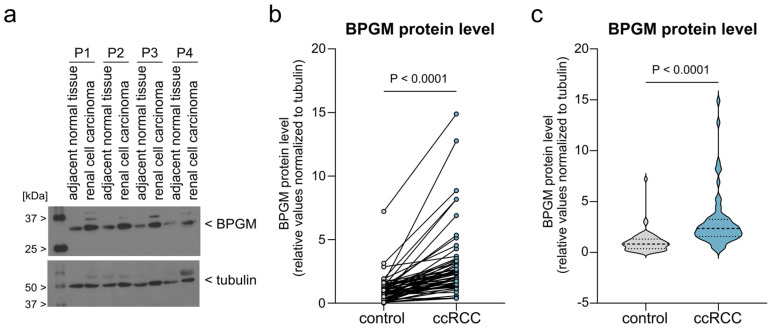
BPGM expression is elevated in human clear cell renal cell carcinoma. (**a**) Representative immunoblot showing BPGM protein levels in ccRCC tumor tissue of four representative patients (P1–4) and matched adjacent normal kidney samples. (**b**) Paired analysis of BPGM protein expression in matched control and tumor samples from individual patients (*n* = 61 paired samples). Each data point represents one biological replicate (individual patient sample). (**c**) Violin plot depicting the distribution of BPGM expression levels in normal kidney tissue and ccRCC samples, illustrating increased expression and interindividual variability in tumor tissue. The dashed line indicates median and dotted lines the interquartile range. Statistical analysis was performed using a paired Student’s *t*-test. Exact *p*-values are indicated in the figure.

To assess the relevance of BPGM in human renal cancer, we first analyzed BPGM expression in ccRCC tumor specimens and matched adjacent normal kidney tissue. Immunoblot analysis revealed markedly higher BPGM protein levels in renal tumor samples compared with corresponding non-malignant tissue (Figure 1a). To account for interindividual variability, BPGM expression was evaluated using paired analysis of matched samples. We observed that the BPGM protein levels were widely increased in ccRCC. Notably, 56 out of 61 samples (91.8%) exhibited at least a 1.5-fold increase in BPGM expression in tumor tissue, and 39 out of 61 samples (63.9%) showed increases exceeding 2-fold. These data indicate that the observed differences are not only consistent in direction but also substantial in magnitude across the majority of cases (Figure 1; Supplementary Figure S1). Visualization of the overall expression distribution further revealed a considerable variation in expression levels across individual samples (Figure 1c).

Together, these data demonstrate that BPGM is robustly and consistently upregulated in human ccRCC, supporting the notion that increased expression of this glycolysis-modulating enzyme represents a characteristic feature of renal tumor tissue.

### 3.2. ccRCC Cell Line A498 Exhibits High Basal BPGM Expression and Only Limited Responsiveness to Epigenetic Stress

**Figure 2:**
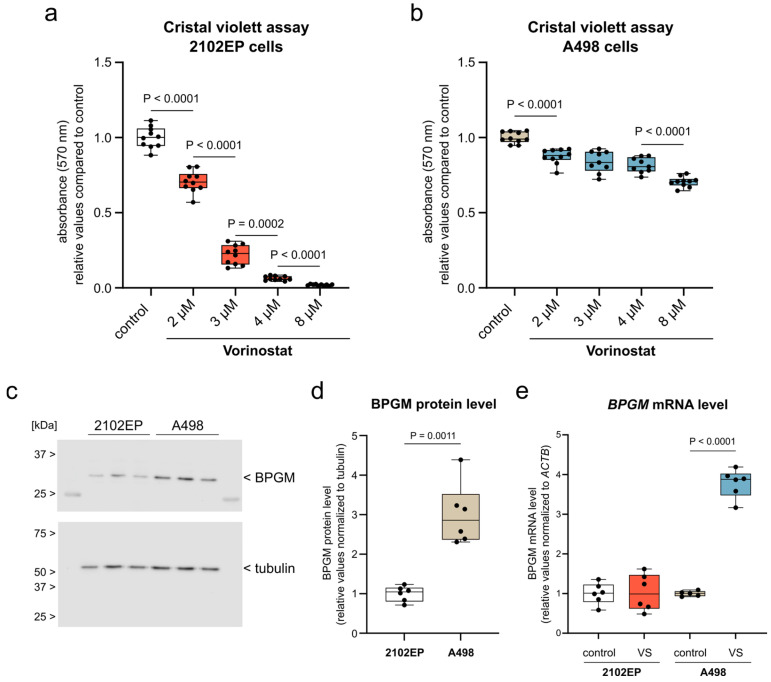
ccRCC cells show limited sensitivity to epigenetic stress and elevated BPGM expression. (**a**,**b**) Crystal violet assays of (**a**) 2102EP and (**b**) A498 cells after 24 h treatment with increasing concentrations of Vorinostat (VS; 2–8 µM). (**a**) *n* = 10 per condition. (**b**) *n* = 10 (control, 2 µM, 8 µM) and *n* = 9 (3 µM, 4 µM; one outlier removed). (**c**,**d**) Basal BPGM protein expression in 2102EP and A498 cells shown as representative immunoblot (**c**) and quantification (**d**) (*n* = 6 per condition). (**e**) *BPGM* mRNA expression after 24 h VS treatment in 2102EP and A498 cells. Expression is shown relative to control. *n* = 6 (2102EP); *n* = 5 (A498 control; one outlier removed) and *n* = 6 (A498 VS). Data are presented as box plots (median with interquartile range; whiskers indicate min–max). Each data point represents one biological replicate. Statistical analysis was performed using Brown–Forsythe ANOVA with Dunnett’s T3 multiple comparisons test (**a**,**b**), Welch’s *t*-test (**d**), and unpaired Student’s *t*-test (2102EP) or Welch’s *t*-test (A498) for (**e**). Details on test selection and normality assessment are provided in Methods. For clarity, only step-wise comparisons along the concentration series are displayed in panels (**a**,**b**). Complete multiple comparisons are provided in Supplementary Figure S2. Color code: white: 2102EP control; beige: A498 control; red: 2102EP + vorinostat; blue: A498 + vorinostat. Exact *p*-values are indicated in the figure.

To enable mechanistic analyses under controlled conditions, we next transitioned from human tumor specimens to an in vitro model of ccRCC. The A498 cell line, which is widely used as a representative ccRCC model, was selected for subsequent experiments. To benchmark epigenetic responsiveness, A498 cells were compared with 2102EP embryonal carcinoma cells, a tumor model known to be highly sensitive to epigenetic modulation [38]. Cells were treated with increasing concentrations of the histone deacetylase inhibitor Vorinostat for 24 h. Under these conditions, 2102EP cells exhibited a pronounced reduction in cell number across the tested concentration range, whereas A498 cells showed markedly reduced sensitivity under identical conditions (Figure 2a,b and Supplementary Figure S2), consistent with their limited responsiveness to epigenetic stress.

We next assessed basal expression of the glycolytic enzyme BPGM in both cell lines. A498 cells displayed substantially higher basal BPGM protein levels compared with 2102EP cells (Figure 2c,d). In addition, Vorinostat treatment for 24 h increased *BPGM* mRNA expression in A498 cells, whereas no comparable induction was observed in 2102EP cells (Figure 2e).

These data establish A498 cells as a suitable in vitro model of ccRCC with limited short-term responsiveness to epigenetic perturbation and elevated basal expression of the glycolytic flux modulator BPGM, providing the basis for subsequent functional analyses.

### 3.3. Loss of BPGM Induces Oxidative Stress and Limits Proliferative Capacity in A498 ccRCC Cells

**Figure 3:**
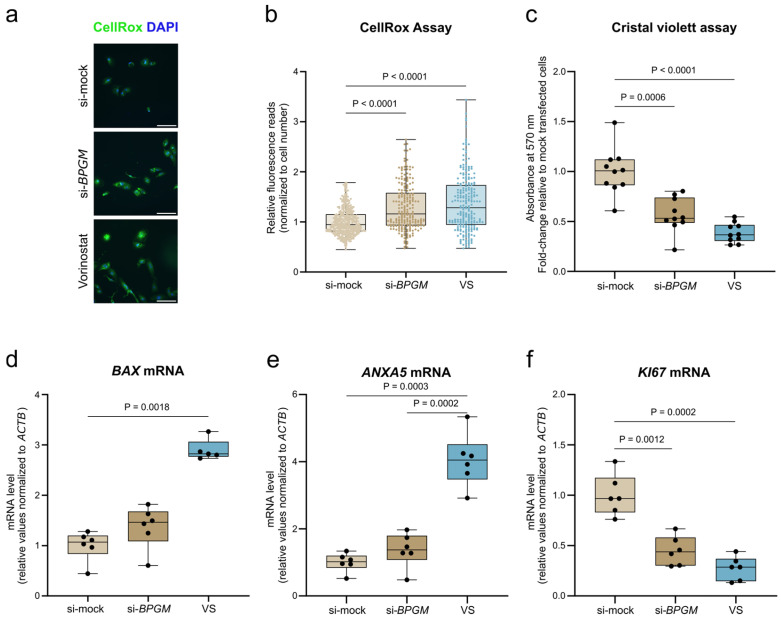
Loss of BPGM induces oxidative stress and limits proliferation in A498 ccRCC cells. (**a**) Representative fluorescence images of CellROX (green) and DAPI (blue) staining in si-mock, si-*BPGM*, and Vorinostat (VS)-treated cells. Scale bar: 100 µm. (**b**) Quantification of CellROX fluorescence intensity normalized to cell number (4 µM VS, 48 h). Data are based on pooled single-cell measurements from two independent experiments (366 si-mock, 232 si-*BPGM*, 224 VS cells). (**c**) Crystal violet assay showing relative cell number (*n* = 10 per condition). (**d**–**f**) qPCR analysis of *BAX* (**d**), *ANXA5* (**e**), and *KI67* (**f**) mRNA expression normalized to *ACTB*. (**d**) *n* = 6 (si-mock, si-*BPGM*) and *n* = 5 (VS; one outlier removed). (**e**,**f**) *n* = 6 per condition. si-mock cells represent non-targeting siRNA with vehicle (DMSO); VS-treated cells were transfected with non-targeting siRNA. Data are presented as box plots (median with interquartile range; whiskers indicate min–max). Each data point represents one biological replicate. Statistical analysis was performed using Kruskal–Wallis test with Dunn’s multiple comparisons test (**b**,**d**) and Brown–Forsythe ANOVA with Dunnett’s T3 multiple comparisons test (**c**,**e**,**f**). Details on test selection are provided in Methods. Exact *p*-values are indicated in the figure.

To define the functional impact of BPGM in ccRCC cells, we examined cellular responses following siRNA-mediated *BPGM* depletion in A498 cells (Figure 3). Efficient knockdown of *BPGM* was confirmed at the mRNA level (Supplementary Figure S3a,b) and protein level (Supplementary Figure S4) prior to functional analyses. Because A498 cells displayed limited responsiveness to epigenetic perturbation after 24 h of Vorinostat exposure in comparative dose–response analyses (Figure 2), subsequent functional experiments were performed using siRNA-mediated *BPGM* knockdown and, for comparison, 4 µM Vorinostat for 48 h to allow the development of measurable phenotypic effects.

Intracellular reactive oxygen species (ROS) levels were assessed using the CellROX assay. *BPGM* silencing significantly increased ROS accumulation compared with mock-transfected control cells (Figure 3a,b), indicating that loss of BPGM is sufficient to induce oxidative stress in ccRCC cells. A comparable increase in ROS was observed following Vorinostat treatment.

To assess the impact on cellular growth, cell number was quantified by crystal violet staining. *BPGM* knockdown resulted in a marked reduction in cell number relative to the control conditions, similar in magnitude to the growth-inhibitory effect observed after Vorinostat treatment (Figure 3c). These findings indicate that loss of BPGM limits the proliferative capacity of A498 cells, supporting a functional role of this metabolic regulator in maintaining cellular growth. To further distinguish between effects on proliferation and apoptosis, expression of apoptosis- and proliferation-associated marker genes was analyzed by quantitative PCR. Vorinostat treatment strongly induced the pro-apoptotic gene *BAX* and increased the *ANXA5* transcript levels, which are frequently elevated in apoptotic cells despite ANXA5 being functionally defined by its membrane localization, whereas *BPGM* silencing alone had no significant effect (Figure 3d,e). In contrast, expression of the proliferation marker *KI67* was significantly reduced following *BPGM* knockdown, comparable to the reduction observed after Vorinostat treatment (Figure 3f).

Collectively, these results demonstrate that loss of BPGM induces oxidative stress and suppresses proliferation in ccRCC cells without triggering a pronounced apoptotic transcriptional response, highlighting a functional distinction between BPGM-associated metabolic regulation and direct pharmacological epigenetic stress despite overlapping phenotypic outcomes.

### 3.4. BPGM Silencing and HDAC Inhibition Elicit Distinct Transcriptional Programs in A498 ccRCC Cells

**Figure 4:**
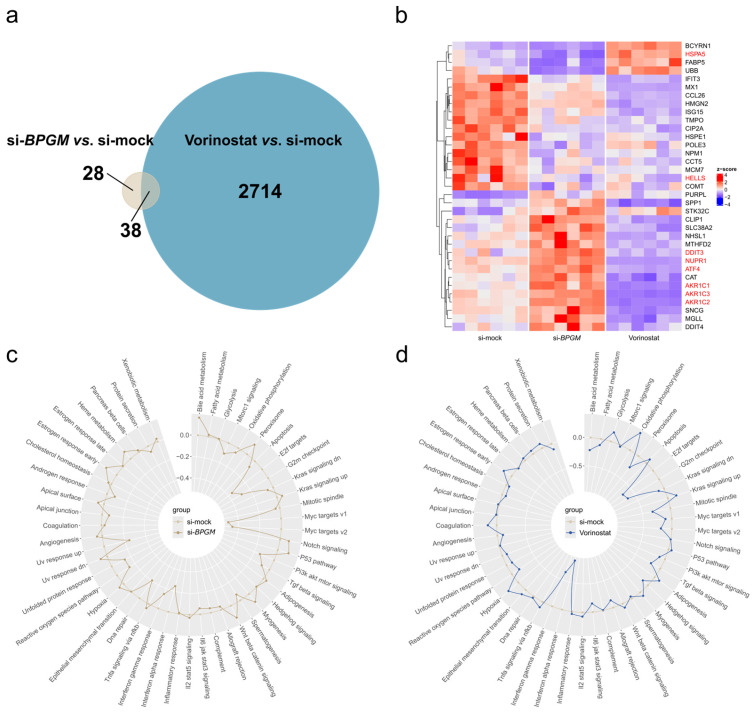
*BPGM* silencing and HDAC inhibition elicit distinct transcriptional programs in A498 ccRCC cells. (**a**) Venn diagram showing the number of differentially expressed genes following siRNA-mediated *BPGM* knockdown or Vorinostat treatment in A498 cells, as well as the overlap between both conditions (*n* = 6 biological replicates per condition). (**b**) Heatmap depicting the expression patterns of genes commonly regulated by *BPGM* silencing and HDAC inhibition, illustrating predominantly inverse regulation between the two perturbations. Genes associated with unfolded protein response and ferroptosis-related pathways are highlighted in red. (**c**,**d**) Pathway-level analyses based on gene set variance analyses showing differential regulation of biological pathways following *BPGM* knockdown (**c**) or Vorinostat treatment (**d**). The differences of the z-scores in relation to control conditions are plotted. Positive values indicate a pathway activation, and negative values indicate a pathway suppression compared to control conditions. Control conditions represent cells transfected with non-targeting siRNA and treated with vehicle (DMSO). Vorinostat-treated cells were transfected with non-targeting siRNA.

To delineate transcriptional programs associated with loss of the metabolic regulator BPGM, we performed transcriptomic profiling of A498 cells and compared these responses with those elicited by HDAC inhibition. Transcriptomic analyses were based on six independent biological replicates per condition. Verification of RNA sequencing data was exemplarily performed by quantitative PCR (Supplementary Figure S3). Differential gene expression analysis revealed a striking disparity in the magnitude of transcriptional changes induced by the two perturbations. While *BPGM* silencing resulted in significant regulation of a limited set of genes, Vorinostat exposure led to widespread transcriptional reprogramming, affecting a substantially larger number of genes (Figure 4a).

Despite producing partially overlapping stress-related phenotypes at the functional level, the transcriptional responses to *BPGM* depletion and HDAC inhibition were largely non-overlapping. Only a small subset of genes was regulated under both conditions, and the majority of these genes displayed opposing regulation following *BPGM* knockdown versus Vorinostat treatment (Figure 4b). This inverse expression pattern indicates that loss of BPGM engages a regulatory program distinct from pharmacological epigenetic reprogramming rather than representing a reduced version of the HDAC inhibitor response.

In sum, our findings demonstrate that *BPGM* depletion elicits a selective and coherent transcriptional response that is clearly separable from the global gene expression changes induced by direct epigenetic intervention. These findings support the concept that intrinsic metabolic regulation shapes stress tolerance in ccRCC independently of widespread epigenetic remodeling.

### 3.5. Pathway-Level Analyses Reveal Selective Stress-Adaptive Signaling upon BPGM Silencing

**Figure 5:**
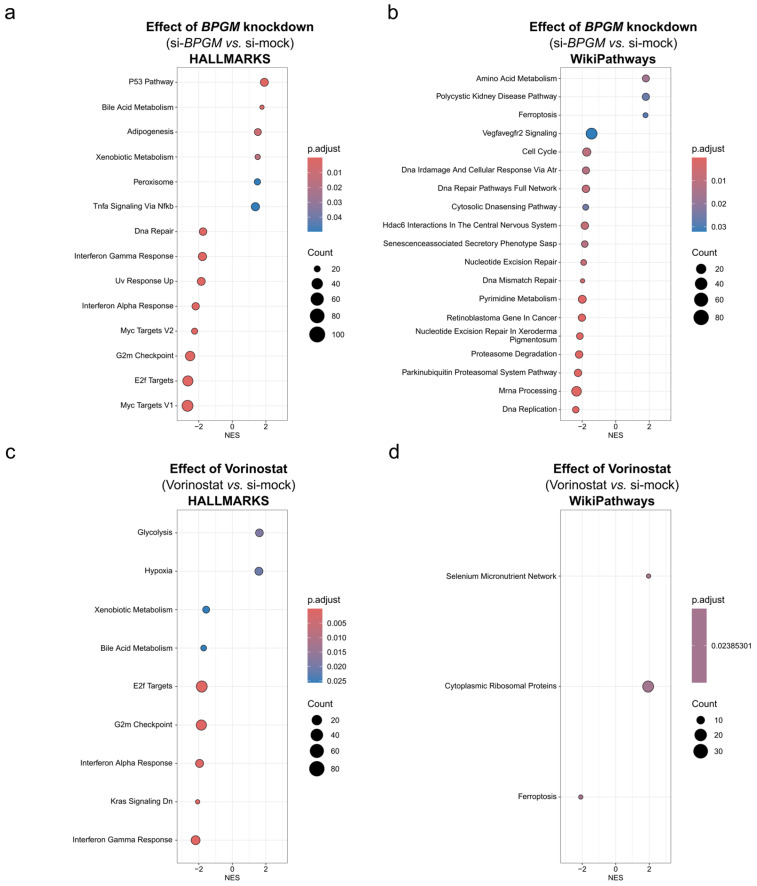
Pathway-level responses to *BPGM* silencing and HDAC inhibition in A498 ccRCC cells. (**a**,**b**) Gene set enrichment analyses (GSEA) showing significantly regulated pathways following siRNA-mediated *BPGM* knockdown in A498 cells, based on the Hallmark (**a**) and WikiPathways (**b**) gene sets (*n* = 6 biological replicates per condition). (**c**,**d**) Gene set enrichment analyses showing significantly regulated pathways following Vorinostat treatment (4 µM, 48 h) in A498 cells, based on the Hallmark (**c**) and WikiPathways (**d**) gene sets (*n* = 6 biological replicates per condition). Pathways were plotted according to their normalized enrichment score (NES), indicating the direction and magnitude of regulation (NES > 0: positive enrichment; NES < 0: negative enrichment). Dot size reflects the number of genes contributing to each pathway, and color indicates adjusted *p*-values derived from GSEA using DESeq2-ranked gene lists. Only significantly enriched pathways are shown. Control conditions represent cells transfected with non-targeting siRNA and treated with the vehicle (DMSO). Vorinostat-treated cells were transfected with non-targeting siRNA to maintain identical transfection conditions across experimental groups.

To extend the gene-level differences observed between *BPGM* depletion and epigenetic perturbation to the pathway scale, we performed gene set enrichment analyses based on the differentially expressed genes identified after *BPGM* knockdown or Vorinostat treatment. Despite the markedly smaller number of regulated genes following *BPGM* silencing, pathway-level analysis revealed a coherent and selective enrichment of stress-associated pathways (Figure 5a,b).

Specifically, *BPGM* depletion preferentially affected pathways linked to cellular stress adaptation, protein homeostasis, metabolic regulation, and cell cycle control. Although the Hallmark p53 pathway showed positive enrichment following *BPGM* silencing, inspection of gene-level expression patterns revealed the selective regulation of stress-adaptive components rather than a coordinated induction of canonical apoptotic genes (Supplementary Figure S3). This observation is consistent with the limited activation of apoptosis markers detected experimentally (Figure 3d,e) and suggests that BPGM loss primarily engages adaptive stress signaling rather than a coherent apoptotic transcriptional program. In contrast, Vorinostat treatment induced a broad and coordinated activation of apoptosis-associated genes (Supplementary Figure S5).

Consistently, unfolded protein response-related and ferroptosis-associated gene sets were prominently enriched under *BPGM* knockdown conditions (Figure 5a,b), whereas these pathways were suppressed upon HDAC inhibition, mirroring the inverse transcriptional patterns observed at the gene level (Figure 4b). By comparison, Vorinostat exposure resulted in broad and heterogeneous pathway alterations—including chromatin regulation, transcriptional control, immune signaling, and metabolic processes (Figure 5c,d)—reflecting the global nature of epigenetic intervention.

Together, these results indicate that *BPGM* depletion engages selective stress-adaptive signaling programs, whereas pharmacologic HDAC inhibition induces widespread and largely non-specific pathway modulation. These pathway-level differences further support the concept that BPGM-centered metabolic perturbation and epigenetic intervention operate at distinct regulatory levels in ccRCC cells.

### 3.6. BPGM Depletion Is Associated with Increased Glycolytic Activity and Epigenetic Regulatory Signatures

**Figure 6:**
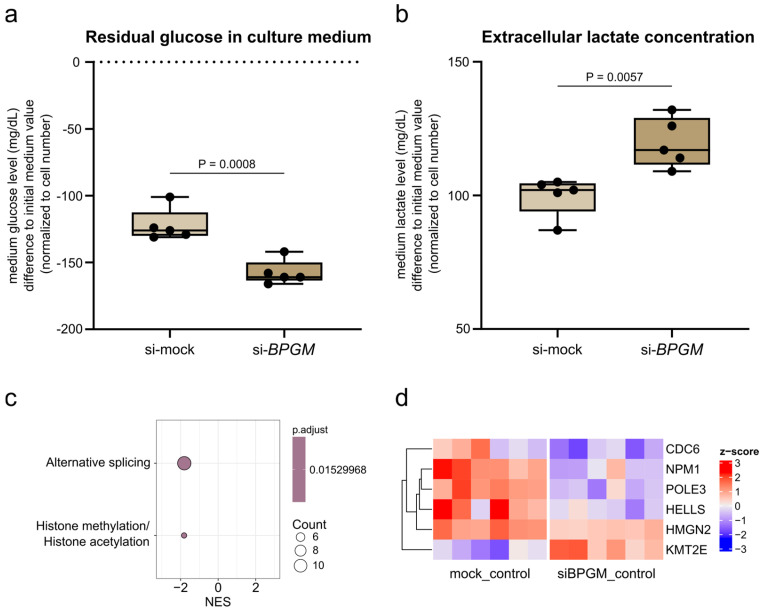
*BPGM* depletion is associated with increased glycolytic activity and modulation of epigenetic regulatory programs in ccRCC cells. (**a**) Residual glucose levels in culture medium after siR-NA-mediated *BPGM* knockdown in A498 cells, shown as difference relative to initial medium values and normalized to cell number (*n* = 5 per condition). The dotted line indicates baseline (0); negative values reflect glucose consumption. (**b**) Extracellular lactate concentrations in culture supernatants normalized to cell number (*n* = 5 per condition). (**c**) Gene set enrichment analysis (GSEA) of differentially expressed genes following *BPGM* knockdown using epigenetic regulator gene sets (*n* = 6 biological replicates per condition). Normalized enrichment scores (NES) indicate direction and magnitude of enrichment. (**d**) Heatmap showing expression changes of selected epigenetic regulators significantly affected by *BPGM* depletion. Expression values are shown as z-scores across samples. Data are presented as box plots (median with interquartile range; whiskers indicate min–max). Each data point represents one biological replicate. Statistical analysis was performed using an unpaired Student’s *t*-test (**a**,**b**). Details on test selection and normality assessment are provided in Methods. Exact *p*-values are indicated in the figure.

To assess whether *BPGM* depletion is associated with alterations in glycolytic activity, we quantified extracellular glucose and lactate levels in A498 cells. *BPGM* knockdown resulted in significantly increased glucose consumption, reflected by reduced residual glucose levels in the culture medium (Figure 6a). This was accompanied by a significant increase in extracellular lactate levels (Figure 6b). Together, these findings are consistent with enhanced glycolytic turnover upon *BPGM* depletion.

To further explore whether BPGM-dependent metabolic perturbation is linked to transcriptional regulatory mechanisms, we performed gene set enrichment analysis using a curated set of epigenetic regulators (EpiFactors database [34,35]). This analysis revealed a significant enrichment of genes associated with histone methylation and acetylation processes following *BPGM* silencing (Figure 6c).

Gene-level inspection identified differential regulation of several chromatin-associated factors, including downregulation of *CDC6*, *NPM1*, *POLE3*, *HELLS*, and *HMGN2*, and upregulation of *KMT2E* (Figure 6d). These genes are functionally linked to chromatin organization, DNA replication, and transcriptional regulation, suggesting that metabolic alterations induced by *BPGM* depletion are accompanied by changes in epigenetic regulatory programs.

Taken together, these data indicate that *BPGM* depletion is associated with a shift in glycolytic activity and concurrent modulation of transcriptional regulatory pathways, providing a potential link between metabolic state and stress-adaptive gene expression in ccRCC cells.

### 3.7. BPGM Depletion Activates ER Stress- and Ferroptosis-Associated Signaling and Promotes Lipid Peroxidation

**Figure 7:**
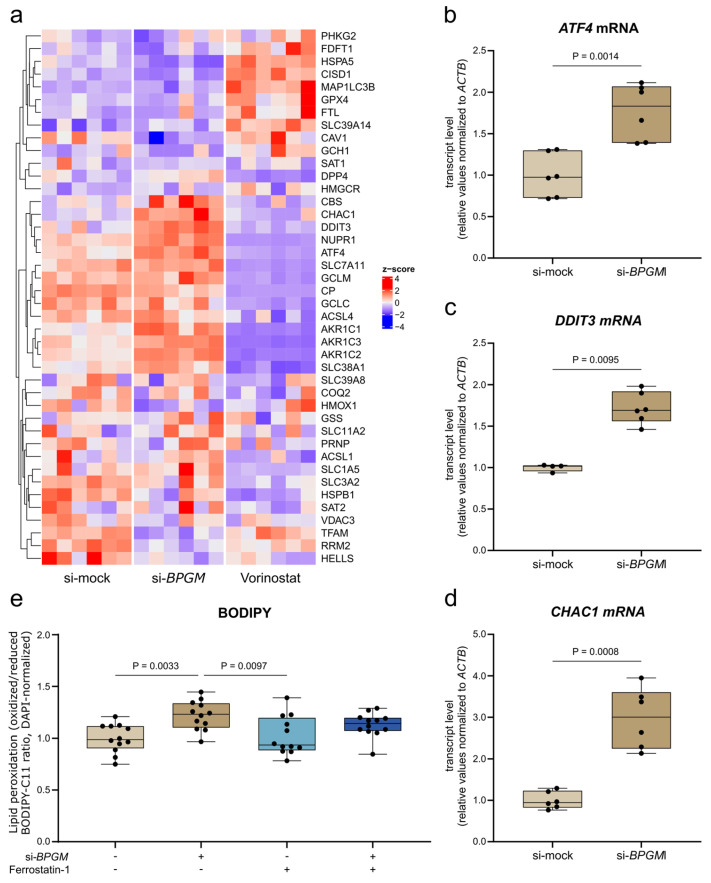
*BPGM* depletion is associated with ER stress-related and ferroptosis-associated signaling and increased lipid peroxidation in A498 ccRCC cells. (**a**) Heatmap of genes associated with endoplasmic reticulum stress, redox regulation, and ferroptosis-related pathways following siRNA-mediated *BPGM* knockdown or Vorinostat treatment (*n* = 6 biological replicates per condition). Expression values are shown as z-scores. (**b**–**d**) qPCR analysis of *ATF4* (**b**), *DDIT3* (**c**), and *CHAC1* (**d**) mRNA expression normalized to *ACTB*. (**b**,**d**) *n* = 6 per condition. (**c**) *n* = 4 (si-mock; two outliers removed) and *n* = 6 (si-*BPGM*). (**e**) Lipid peroxidation assessed by BODIPY-C11 staining, expressed as ratio of oxidized to reduced fluorescence and normalized to DAPI intensity (*n* = 12 per condition). Data are presented as box plots (median with interquartile range; whiskers indicate min–max). Each data point represents one biological replicate. Statistical analysis was performed using Welch’s *t*-test (**b**,**d**), Mann–Whitney U test (**c**), and one-way ANOVA with Tukey’s multiple comparisons test (**e**). Details on test selection and normality assessment are provided in Methods. Exact *p*-values are indicated in the figure.

To further characterize the stress-adaptive pathways selectively engaged upon *BPGM* depletion, we examined the expression of genes associated with endoplasmic reticulum (ER) stress and ferroptosis. Transcriptomic analysis revealed the coordinated regulation of multiple genes involved in these pathways following *BPGM* knockdown (Figure 7a). Notably, components of the ATF4–CHOP–CHAC1 axis were among the most prominently induced genes.

These findings were validated at the transcriptional level by quantitative PCR. *BPGM* silencing significantly increased mRNA expression of *ATF4*, *DDIT3* (CHOP), and *CHAC1* compared with mock-transfected control cells (Figure 7b–d), consistent with the activation of ER stress-associated signaling.

To assess whether *BPGM* depletion is accompanied by increased lipid peroxidation, a ferroptosis-associated feature, cells were stained with the lipid peroxidation sensor BODIPY-C11. *BPGM* knockdown led to a significant increase in the ratio of oxidized to reduced BODIPY fluorescence, consistent with enhanced lipid peroxidation (Figure 7e). Treatment with the ferroptosis inhibitor Ferrostatin-1 attenuated lipid peroxidation in *BPGM*-silenced cells, although levels were not fully restored to control conditions. The persistence of elevated lipid peroxidation after ferrostatin treatment, even following normalization to cell number, suggests that BPGM loss induces a broader redox imbalance rather than a purely ferroptosis-driven process.

These results demonstrate that loss of BPGM engages ER stress-associated ferroptosis-related signaling and promotes lipid peroxidation in ccRCC cells, supporting a link between glycolytic flux perturbation and lipid peroxidation-associated stress response.

## 4. Discussion

In this study, we examined whether the modulation of glycolytic flux through 2,3-bisphosphoglycerate mutase (BPGM) contributes to stress tolerance and transcriptional adaptation in clear cell renal cell carcinoma (ccRCC). By integrating analyses of human ccRCC specimens, targeted perturbation of BPGM, and transcriptomic profiling, we identified BPGM as a metabolic regulator associated with redox control and cellular stress resilience. Although both BPGM depletion and histone deacetylase inhibition induced oxidative stress and growth suppression, they elicited fundamentally distinct transcriptional and pathway-level responses, indicating that intrinsic metabolic properties shape how ccRCC cells accommodate cellular stress.

Our findings extend the concept of ccRCC as a metabolically resilient tumor entity. ccRCC is characterized by profound metabolic reprogramming, including enhanced glycolysis and altered redox homeostasis, which together support tumor cell survival under fluctuating environmental conditions [39,40,41]. In this context, loss of functional VHL, a defining feature of ccRCC, leads to constitutive stabilization of hypoxia-inducible factors (HIFs) and promotes a glycolysis-prone metabolic phenotype accompanied by redox imbalance [42,43,44]. Within this context, BPGM represents a distinct regulatory node, as it introduces a dedicated glycolytic shunt via the production of 2,3-bisphosphoglycerate and thereby modulates the distribution of glycolytic intermediates. While BPGM has long been studied in erythrocytes for its role in regulating hemoglobin oxygen affinity, its function outside this context has only recently gained attention. In renal tubular cells, BPGM was shown to protect against oxidative stress and apoptosis [18], and our data suggest that a similar principle applies in renal cancer cells.

Earlier biochemical studies from the 1970s through to the 1990s investigated the Rapoport–Luebering shunt in a variety of tumor entities by assessing 2,3-bisphosphoglycerate mutase and phosphatase activities, which are both catalyzed by BPGM. In several solid tumors such as brain, lung, colon, and liver carcinomas, these analyses revealed a paradoxical pattern: while classical glycolytic enzymes such as phosphoglycerate mutase and enolase were frequently elevated, activities linked to 2,3-bisphosphoglycerate metabolism were reduced compared with corresponding normal tissues ([45], and references therein). These findings led to the interpretation that many tumors suppress the energetically inefficient Rapoport–Luebering shunt despite high overall glycolytic flux. Importantly, these earlier studies also highlighted substantial tissue specificity and heterogeneity and did not include renal tissue or renal cell carcinoma. Against this background, our data suggest that ccRCC represents a distinct metabolic context in which sustained BPGM expression and the modulation of glycolytic flux contribute to stress tolerance rather than ATP optimization. Consistent with the high basal expression of BPGM in the distal tubular epithelium of the kidney [18], our findings raise the possibility that sustained BPGM levels represent a renal lineage-associated metabolic trait retained in ccRCC. Such a kidney-specific metabolic context may distinguish ccRCC from other tumor entities in which the Rapoport–Luebering shunt is reduced and could contribute to the characteristic stress resilience of renal cancer cells.

Accumulating evidence indicates that metabolic cues can influence epigenetic regulation by modulating the activity of chromatin-modifying enzymes in a context-dependent manner [46]. Histone deacetylase inhibitors themselves are known to affect metabolic pathways and redox regulation across multiple tumor entities, including mitochondrial function and ferroptosis-related processes [47,48]. However, in the present ccRCC model, direct pharmacological HDAC inhibition elicited broad transcriptional responses that only partially overlapped with those induced by *BPGM* depletion. The limited overlap and predominantly inverse regulation of genes affected by the two perturbations therefore argue against a simple linear coupling between metabolic and epigenetic stress responses in this context and instead are consistent with a model in which BPGM-associated metabolic states establish a permissive framework shaping cellular stress adaptation.

While the inverse regulation of shared genes observed between *BPGM* depletion and HDAC inhibition suggests fundamentally distinct transcriptional responses, we acknowledge that such patterns could, in principle, also reflect differences in stress magnitude or temporal dynamics rather than entirely distinct regulatory programs. However, the comparable reduction in cell number across conditions argues against major differences in overall stress intensity and supports the interpretation that metabolic and epigenetic perturbations engage qualitatively different transcriptional responses. It should be noted that differences in responsiveness to HDAC inhibition may also reflect cell line-specific response kinetics rather than absolute resistance, which may contribute to the observed effects.

An alternative, but not mutually exclusive, interpretation of our data is that changes in glycolytic intermediate routing following *BPGM* depletion may amplify metabolite-driven epigenetic regulation rather than bypass it. In this scenario, BPGM loss may promote glycolysis-linked epigenetic adaptation, whereas HDAC inhibition targets chromatin regulation more directly and globally, resulting in opposing transcriptional responses. From this perspective, the inverse gene expression patterns observed upon *BPGM* depletion and Vorinostat treatment are not unexpected but instead underscore fundamentally different modes by which metabolic versus pharmacological perturbations interface with epigenetic regulation. In line with this concept, *BPGM* depletion was associated with the differential regulation of genes linked to epigenetic control, including the upregulation of *KMT2E* and downregulation of several chromatin-associated factors. Notably, this transcriptional pattern coincided with the reduced enrichment of E2F target genes and decreased cell proliferation. KMT2E has been implicated in the chromatin-associated regulation of transcriptional programs linked to cell cycle control and differentiation, including the modulation of H3K4 methylation-dependent processes [49,50]. While the precise mechanistic role of KMT2E in this context remains to be defined, the observed expression pattern is consistent with a shift away from E2F-driven proliferative programs toward a more regulated or differentiation-associated transcriptional state. This concept is further supported by recent work demonstrating that BPGM shapes NFAT5-driven cellular responses in an independent experimental context [51]. Notably, also in this setting, the link between metabolic perturbation and transcriptional regulation is supported by indirect evidence, underscoring the emerging but still unresolved nature of this connection. Viewed in this light, histone deacetylase inhibition targets an appropriate regulatory layer to interfere with stress-adaptive transcriptional programs; however, the opposing transcriptional outcomes suggest that pharmacological HDAC blockade may not fully recapitulate or disrupt metabolically defined states that underpin stress tolerance in ccRCC. Instead, modulation of glycolytic flux may represent a distinct and potentially more selective axis influencing tumor resilience and treatment insensitivity.

A prominent feature selectively associated with *BPGM* depletion was an increase in lipid peroxidation and increased expression of ER stress-associated genes, including components of the ATF4–CHOP–CHAC1 axis, which have been linked to ferroptosis through the regulation of glutathione metabolism and redox balance [52,53,54]. ccRCC cells have been reported to exhibit an intrinsic susceptibility to ferroptosis due to lipid accumulation and altered redox homeostasis while simultaneously engaging antioxidant programs that restrain full ferroptotic execution [55]. Within this framework, the transcriptional changes observed after *BPGM* depletion are consistent with a shift toward a lipid peroxidation-prone state rather than induction of a fully executed ferroptotic program. In contrast, ferroptosis-associated signatures were suppressed upon HDAC inhibition in A498 cells despite the induction of oxidative stress, highlighting that the engagement of lipid peroxidation pathways is not a generic consequence of cellular stress but depends on the underlying metabolic context.

Beyond identifying BPGM as a stress-associated metabolic regulator, our findings also invite a broader interpretation of glycolytic reprogramming in cancer. The Warburg effect is traditionally viewed as a shift toward aerobic glycolysis to support energy production and biosynthetic demands. Our data suggest that this paradigm may be extended by considering the glycolytic flux distribution and accumulation of specific intermediates as regulatory variables in tumor cell survival. In this context, modulation of glycolytic flux, e.g., via BPGM, may indirectly influence transcriptional regulation by shaping the availability of metabolites that act as cofactors or modulators of chromatin-modifying enzymes, thereby contributing to the epigenetic landscape.

An additional aspect emerging from our analysis is the pronounced inter-individual variability in BPGM expression observed in human ccRCC specimens. A similarly broad range of BPGM abundance has previously been reported in human erythrocytes, indicating that BPGM expression is subject to considerable physiological heterogeneity [56]. Such variability may influence individual redox buffering capacity and stress tolerance, potentially contributing to divergent tumor responses, although this study was not designed to address patient stratification nor treatment outcome.

Several limitations should be acknowledged. Our functional analyses were primarily performed in a single ccRCC cell line, and the molecular intermediates linking glycolytic flux to transcriptional regulation remain unresolved. However, the observation that BPGM depletion induces comparable stress responses in independent cellular systems [18] suggests that the underlying mechanism may not be restricted to ccRCC but could reflect a more general feature of BPGM-expressing cells. In particular, the proposed link between metabolic perturbation and epigenetic regulation is currently supported by indirect evidence and warrants further mechanistic investigation.

## 5. Conclusions

This study identified BPGM as a previously underappreciated metabolic regulator linked to stress adaptation in clear cell renal cell carcinoma. By integrating analyses of human tumor specimens with functional and transcriptomic data, we show that the perturbation of BPGM is associated with altered glycolytic activity, redox imbalance, and distinct stress-adaptive transcriptional responses that differ from those induced by epigenetic intervention.

While the mechanistic connection between glycolytic flux and transcriptional regulation remains to be fully resolved, our findings support the concept that the metabolic state contributes to shaping cellular stress responses in ccRCC. In this context, BPGM emerges as part of a metabolic framework that may influence tumor resilience and treatment responsiveness.

Future studies will be required to define the underlying molecular mechanisms and to establish direct links between metabolic intermediates and chromatin regulation. In particular, the development of sensitive and robust analytical approaches for quantifying low-abundance metabolites such as 2,3-bisphosphoglycerate in non-erythroid cells represents an important technical challenge that will be critical for advancing this field.

## Data Availability

RNA sequencing data generated in this study have been deposited in the Gene Expression Omnibus (GEO) under accession number GSE319257. Other data supporting the findings of this study are available from the corresponding author upon reasonable request.

## Supplementary Materials

The following supporting information can be downloaded at: https://www.mdpi.com/article/10.3390/cells15070633/s1, Table S1: Primer sequences for qPCR; Table S2: Detailed settings for ORA analysis; Figure S1: Individual BPGM protein expression levels in paired ccRCC and adjacent normal kidney tissue samples; Figure S2: Complete statistical comparisons for the crystal violet assay shown in Figure 2a,b; Figure S3: Validation of RNA sequencing data by quantitative PCR; Figure S4: Validation of *BPGM* knockdown at the protein level in A498 cells; Figure S5: Gene-level expression patterns of apoptosis- and p53-associated pathways following *BPGM* silencing or Vorinostat treatment in A498 cells. Uncropped Western blot figures.

## Abbreviations

The following abbreviations are used in this manuscript:

ANOVA	Analysis of Variance
ANXA5	Annexin A5
ATF4	Activating Transcription Factor 4
BAX	Bcl-2-Associated X Protein
2,3-BPG	2,3-Bisphosphoglycerate
BPGM	2,3-Bisphosphoglycerate Mutase
ccRCC	Clear Cell Renal Cell Carcinoma
CHAC1	ChaC Glutathione Specific Gamma-Glutamylcyclotransferase 1
CPA4	Carboxypeptidase A4
DAPI	4′,6-Diamidino-2-Phenylindole
DESeq2	Differential Expression Sequencing 2
DGE	Differential Gene Expression
DMSO	Dimethyl Sulfoxide
ER	Endoplasmic Reticulum
GSEA	Gene Set Enrichment Analysis
GSVA	Gene Set Variation Analysis
HDAC	Histone Deacetylase
HDACi	Histone Deacetylase Inhibitor
HIF	Hypoxia-Inducible Factor
NES	Normalized Enrichment Score
NGS	Next-Generation Sequencing
NUPR1	Nuclear Protein 1
qPCR	Quantitative Polymerase Chain Reaction
ROS	Reactive Oxygen Species
siRNA	Small Interfering RNA
T3	Dunnett’s T3 Test
UPR	Unfolded Protein Response
UMI	Unique Molecular Identifier
