# Supplementary material for: 2,3-Bisphosphoglycerate Mutase (BPGM), a Metabolic Player Shaping Stress-Adaptive Transcriptional States in Clear Cell Renal Cell Carcinoma
Source: Cells. 2026 Mar 31;15(7):633. doi: 10.3390/cells15070633 (PMC13072826; doi:10.3390/cells15070633)
Supplement: Supplementary file 1 [file cells-15-00633-s001.zip › cells-4194084-supplementary.pdf]

SUPPLEMENT

2,3-Bisphosphoglycerate mutase (BPGM), a metabolic player shaping stress-adaptive transcriptional states in clear cell renal cell carcinoma

Philipp N. Becker, Vera A. Kulow, Claudia S. Czopek, Kameliya Roegner, Gohar Ter-Avetisyan, Anica Loth, Bianca Nitzsche, Cem Erdogan, Adrian Schreiber, Michael Höpfner, Michael Fähling*, Robert Labes

* Corresponding author: Michael Fähling, Charité – Universitätsmedizin Berlin, Institut für Translationale Physiologie, Charitéplatz 1, D-10117 Berlin, Germany. michael.faehling@charite.de

Supplementary Table S1: Primer sequences for qPCR.

Target gene	Primer Sequence (5′ to 3′)	
BPGM	Forward:	CAA CTG CCA CGG TCG GAA AG
	Reverse:	TGC TTG GAT CGC CTC TTG GT
BAX	Forward:	ACA GGG GCC CTT TTG CTT CAG
	Reverse:	GCT CCC GGA GGA AGT CCA AT
ANXA5	Forward:	AAC AGA GAC CCT GAT GCT GGA
	Reverse:	TTG CCA GAA GTC TCG CGG TC
KI67	Forward:	CGG ATC GTC CCA GTG GAA G
	Reverse:	TTG CCT CCT GCT CAT GGA TT
ATF4	Forward:	GGG AGT TGG CTT CTG ATT CTC A
	Reverse:	AGA AGG CAT CCT CCT TGC TG
DDIT3	Forward:	AGA GCT GGA ACC TGA GGA GAG A
	Reverse:	TGC AGG ATA ATG GGG AGT GGC
CHAC1	Forward:	GGA TTT TCG GGT ACG GCT CC
	Reverse:	CAC ACG GCC AGG CAT CTT
CPA4	Forward:	GGG GCC CTT ATT GGG TCC AG
	Reverse:	CAG GAC ATC CAC AGG CCG AT
NUPR1	Forward:	CTC GGA GGT GGA GGC CG
	Reverse:	TGG AGG CGC TTT CCT GGT AG
ACTB	Forward:	TGA AGT GTG ACG TGG ACA TC
	Reverse:	GTC ATA GTC CGC CTA GAA GC

Supplementary Table S2: Detailed settings for GSEA analysis.

**Setting**	**Readable name**	**Value**
minGSSize	Minimum gene set size	10
maxGSSize	Maximum gene set size	500
pvalueCutoff	adjusted p-value cutoff on enrichment tests to report as significant	< 0.05
pAdjustMethod	Method used for adjusting p-value for multiple testing	BH

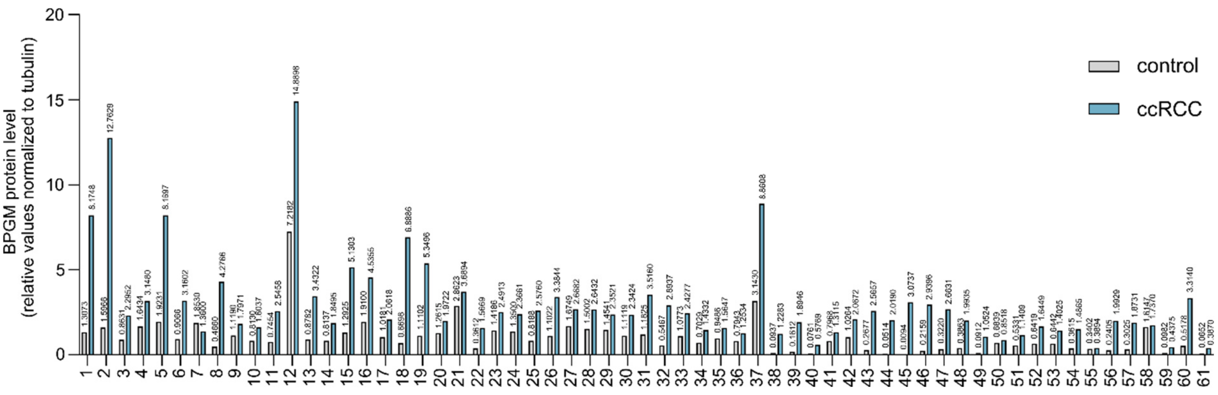

Supplementary Figure S1. Individual BPGM protein expression levels in paired ccRCC and adjacent normal kidney tissue samples.

Bar plot showing BPGM protein expression levels for all individual patient samples (n = 61), quantified by densitometric analysis of Western blot signals normalized to tubulin. For each patient, matched tumor (ccRCC) and corresponding adjacent normal kidney tissue samples were analyzed in a paired manner. Samples were processed on the same gel, and a pooled reference sample was included across experiments to enable inter-gel normalization. This representation illustrates the distribution and variability of BPGM expression across the cohort and confirms the overall shift toward higher BPGM levels in ccRCC compared with normal tissue.

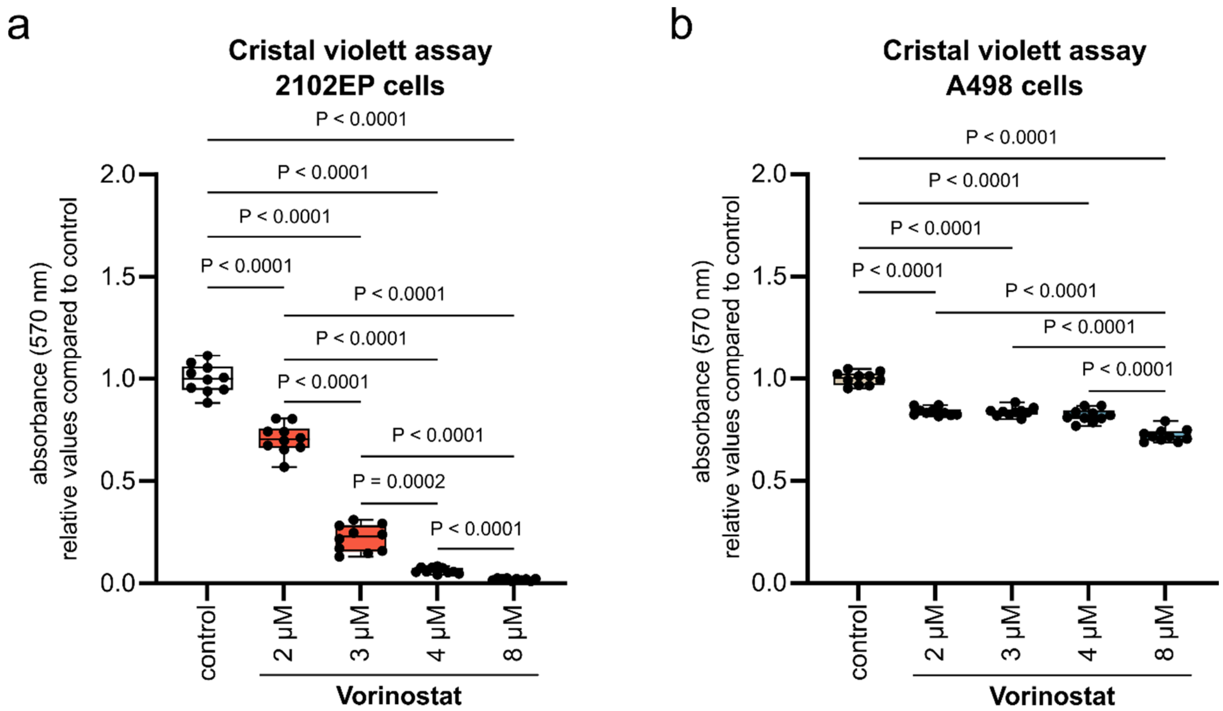

Supplementary Figure S2. Complete statistical comparisons corresponding to the crystal violet assays shown in Figure 2a and b.

All pairwise group comparisons are displayed. Data are based on biological replicates as indicated in Figure 2 (n = 10 per condition for Figure 2a; n = 10 for control, 2 µM, and 8 µM, and n = 9 for 3 µM and 4 µM in Figure 2b, with one outlier removed based on ROUT test). Statistical analysis was performed based on data distribution and variance. Normality was assessed using the Kolmogorov–Smirnov test. Brown–Forsythe and Welch ANOVA followed by Dunnett's T3 multiple comparisons test were applied.

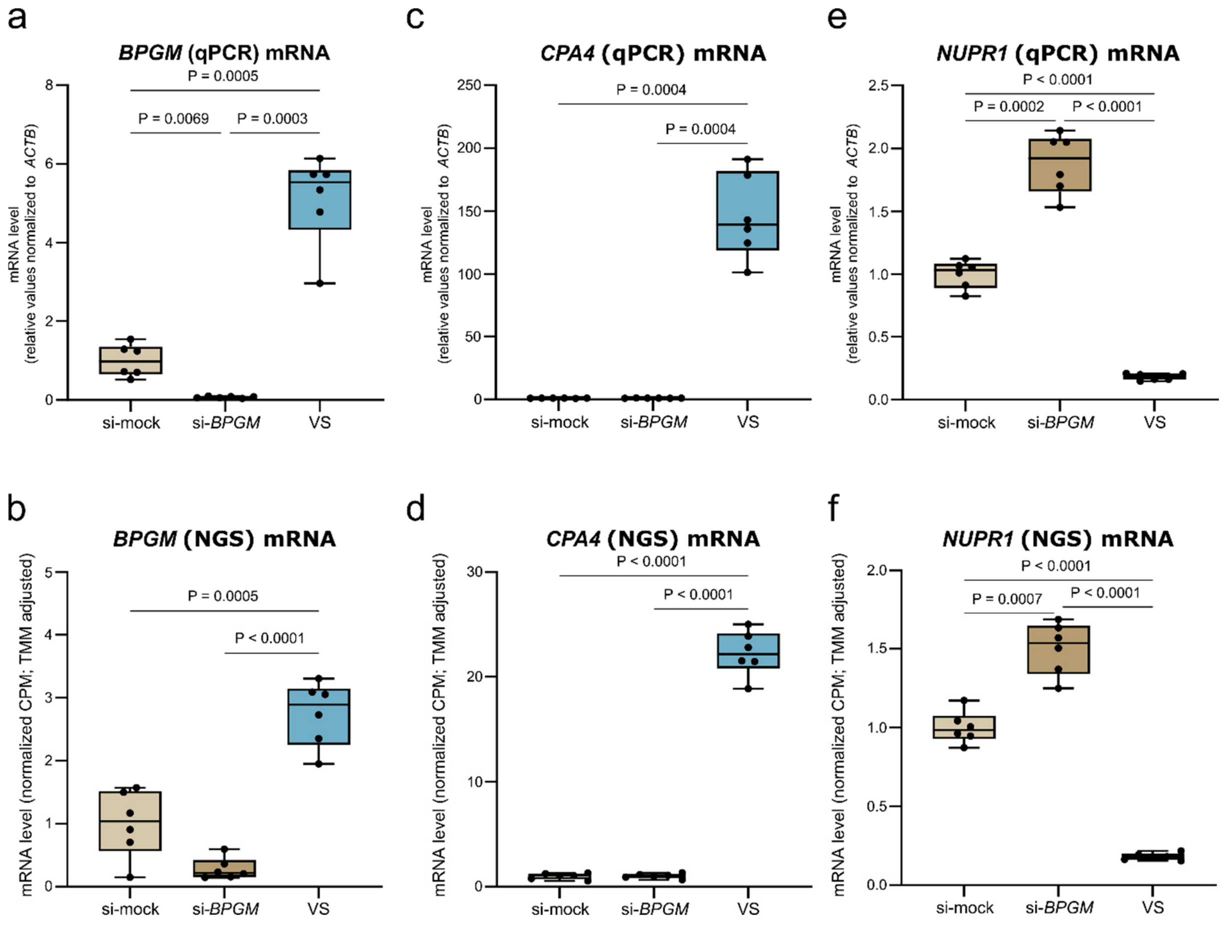

Supplementary Figure S3. Validation of RNA sequencing data by quantitative PCR.

Relative mRNA expression levels of *BPGM*, *CPA4*, and *NUPR1* were assessed by quantitative PCR (qPCR; panels a, c, e) and compared with normalized RNA sequencing read counts (panels b, d, f) in A498 cells following siRNA-mediated *BPGM* knockdown or Vorinostat (VS) treatment (n = 6 biological replicates per condition). *CPA4* was selected as a representative gene induced by histone deacetylase inhibition, whereas *NUPR1* represents a stress-responsive gene regulated upon *BPGM* depletion. qPCR results are consistent with the direction and magnitude of gene regulation observed in the RNA sequencing dataset. Data are shown as box plots with individual data points representing biological replicates. qPCR measurements were performed in technical triplicates and averaged prior to analysis. Statistical analysis was performed based on data distribution and variance. Normality was assessed using the Kolmogorov–Smirnov test. Brown–Forsythe and Welch ANOVA followed by Dunnett's T3 multiple comparisons test were applied for panels (a–f). Exact p-values are indicated in the figure.

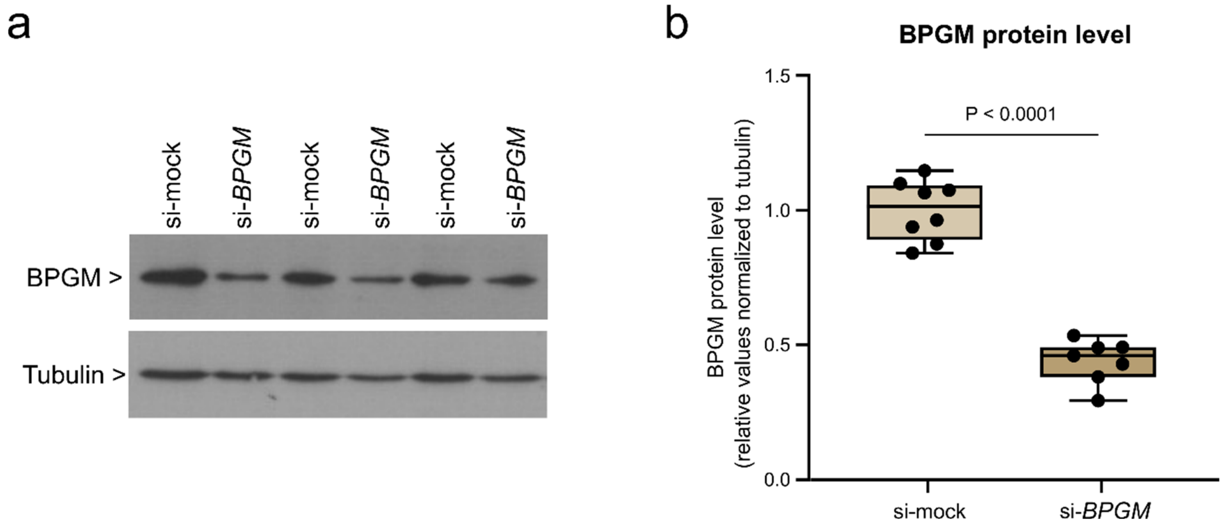

Supplementary Figure S4. Validation of *BPGM* knockdown at the protein level in A498 cells.

(a) Representative Western blot showing BPGM protein expression following siRNA-mediated knockdown (si-*BPGM*) compared with control cells (si-mock) after 48 h. Tubulin was used as a loading control. (b) Quantification of BPGM protein levels normalized to tubulin. Data represent independent biological replicates (si-mock: n = 8; si-BPGM: n = 7). In the si-*BPGM* group, one value was identified as an outlier using the ROUT method (Q = 1%) and excluded from the analysis. Data are presented as box plots showing the median with lower and upper quartiles; whiskers indicate minimum and maximum values. Statistical analysis was performed based on data distribution. Normality was assessed using the Kolmogorov–Smirnov test, and an unpaired Student's t-test was applied. Exact p-values are indicated in the figure.

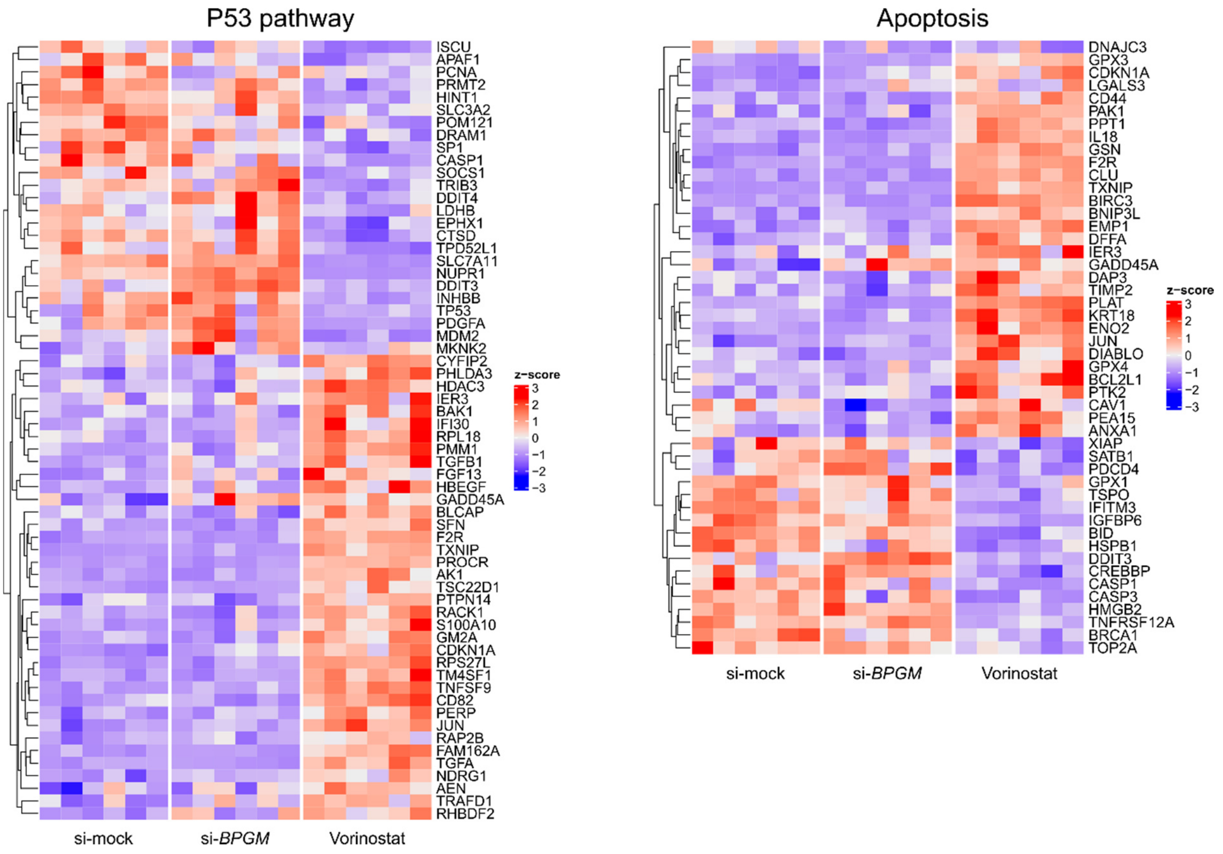

Supplementary Figure S5. Gene-level expression patterns of apoptosis- and p53-associated pathways following *BPGM* silencing or Vorinostat treatment in A498 cells.

Heatmaps depict normalized expression of genes belonging to the Hallmark "p53 pathway" and "Apoptosis" gene sets derived from RNA sequencing data (n = 6 biological replicates per condition). Rows represent individual genes and columns represent experimental conditions (si-mock, si-*BPGM*, Vorinostat). Color scale indicates relative expression changes after variance-stabilizing transformation (DESeq2). Vorinostat treatment was associated with a broad and coordinated transcriptional response across apoptosis-related genes, whereas *BPGM* silencing resulted in more selective regulation of stress-associated components without a uniformly coordinated induction of canonical apoptotic genes. These data complement the pathway-level analyses shown in Figure 5.
